# User demands analysis of Eco-city based on the Kano model—An application to China case study

**DOI:** 10.1371/journal.pone.0248187

**Published:** 2021-03-10

**Authors:** Jinqiu Li, Qingqin Wang, Yitong Xuan, Hao Zhou

**Affiliations:** 1 School of Civil Engineering, Chongqing University, Chongqing, China; 2 Beijing Tsinghua Tongheng Urban Planning & Design Institute, Beijing, China; 3 Key Laboratory of Eco Planning & Green Building, Ministry of Education, Tsinghua University, Beijing, China; 4 China Academy of Building Research, Beijing, China; 5 Think Tank Research Center, Tsinghua University, Beijing, China; University of Defence in Belgrade, SERBIA

## Abstract

Eco-cities have witnessed rapid growth in these years worldwide. As the Eco-cities entering operation stage gradually, more and more researchers have found that users (who are living or working in the Eco-cities) satisfaction is one of the most important factors to determine the success or failure of Eco-cities. Therefore, it is very important to investigate the user demands to attract more citizens willing to live or work in the Eco-cities, which will make the development of Eco-cities more sustainable and solid. The recent researches on user demands investigation and analysis in the Eco-cities mainly focused on understanding the user need itself, yet lack of research on the relationship between the user demand and user satisfaction. This paper initially introduced the Kano model analysis method to the research field of user demands in Eco-city, to explore the relationship between the user demand and user satisfaction. After proposing user demands library in Eco-city (including Land use, Ecological environment, Green building, Energy utilization, etc.), the user demands classification and importance analysis methods of Eco-city were proposed based on Kano model. The questionnaire survey for users of two Eco-cities in China as case study was conducted, consisted of user demand items questionnaire based on the Kano model and a questionnaire on the importance of the user demand items. By utilizing the integration of quantitative analysis methods based on the Kano model and Analytic Hierarchy Process (AHP) method, the final ranking of user demands importance was obtained. Comparing with the existing literatures in terms of user demands research for Eco-city, the user demands analysis method based on Kano model of this paper, is able to reveal the influence degree of user satisfaction towards the facilities and services provided in the Eco-city. The user demands analysis method can be used for other researchers worldwide to investigate and quantitively analyze user demands according to their local development situation and preference of Eco-city. The user demands analysis results obtained through this method, can benefit different stages of Eco-city.

## 1. Introduction

Eco-cities, which pour effort into eliminating the overall carbon footprint of the city whilst helping the humans and the nature to coexist aiming towards sustenance and sustainability [1]. Eco-cities have witnessed rapid growth in these years worldwide [2,3]. As the Eco-cities entering operation stage gradually, more and more researchers have found that users (who are living or working in the Eco-cities) satisfaction is one of the most important factors to determine the success or failure of Eco-cities. Kitakyushu eco-city is recognized the most mature model Eco-city project in Japan. The KPIs (key performance indicators) of this project are considered successful based on the quantified data of residents’ satisfaction degree, which indeed improved conditions for the aging population of this city [4]. Another well-known example of Eco-city is Sino-Singapore Tianjin Eco-city (SSTEC) of China, which generally accepted as the flagship project in China and one of the most successful Eco-cities in the world [5,6]. The construction and operation efforts of this Eco-city have been mainly put into aspects which residents able to truly experience, such as natural environment, man-made environment and life style [4,7]. By contrast, one of the key reasons why Masdar city (Abu Dhabi, UAE) and Dongtan city (Shanghai, China) considered as failure projects, is that both of them placed too much emphasis on realization of zero-carbon, and mandated the residents to adjust with lesser ‘comforts’ than they are used to [8,9]. Therefore, it is very important to investigate the user demands to attract more citizens willing to live or work in the Eco-cities, which will make the development of Eco-cities more sustainable and solid.

Caprotti et al. [10] carried out fifteen interviews with the residents in Sino-Singapore Tianjin Eco-city of China in terms of the lived experiences, and found that social sustainability need to be paid more attention. Liu et al. [11] utilized Structural Equation Model (SEM) to investigate the residents’ repurchase intention of Green Residential Building (GRB) in Eco-city of China. Marciniak et al. [12] conducted structural interviews with the visitor perception of informal green spaces in Poland and concluded that informal green spaces are important complementary to formal green spaces in the city. To examine residents’ WTP (willingness to pay) for GRBs (green residential buildings) and its determinants, Liu et al. [13] conducted a survey among 511 current GRB occupants living in Sino-Singapore Tianjin Eco-city in China, and latent class regression was used to analyze the heterogeneity of their preferences. Sun et al. [14] collected the social media data, by applying the artificial intelligence technique to analyze the visitor responses to the green and open spaces in Shenzhen, China. Friederike et al. [15] conducted a quantitative questionnaire survey among people aged 50 years and older throughout the city of Berlin, to explore the older people’s urban green space visitation patterns. Erik et al. [16] used a discrete choice experiment to explore people’s preferences and willingness to pay for green features in an urban Neighborhood Management development zone in Berlin. Monteiro et al. [17] investigated the citizen demand of sustainability for urban forests based on i-Tree Eco surveys.

The above recent researches on user demands investigation and analysis in the Eco-cities mainly focused on understanding the user need itself, yet lack of research on the relationship between the user demand and user satisfaction. Because improving the user (citizen who lives or works in the Eco-city) satisfaction will be the ultimate purpose of construction and operation of the Eco-city, yet user demands survey is just the process. Actually, the classification of user demands are various, same efforts to different classification of user demands may lead to different degree of satisfaction. In other words, different classification of user demands in Eco-city may need different strategies to satisfy. Therefore, an analysis method on revealing the relationship between user demands and user satisfaction was initially introduced to research field of Eco-city in this paper. The Kano model is a famous and important theoretical and quantitative model for the research of customer satisfaction towards product quality attribute [18] for different industries. Aliyu et al. [19] utilized integration method of Kano model and quality function deployment (QFD) to investigate the user demands for sport earphone. Wu et al. [20] conducted a study adopted Kano two-dimensional quality model to investigate the users’ needs on twenty service attributes of rehabilitation buses. Juan et al. [21] exploring sustainable planning strategies for public housing in Taiwan, the Kano model was adopted as a theoretical base. Ma et al. [22] used Kano model to differentiate between future vehicle-driving services demands. Xu et al. [23] presented a requirements analysis method based on Fuzzy Kano Model, which to improve the quality of virtual reality interior design software. Yao et al. [24] conducted the Kano model analysis of features for mobile security applications to gain more customer satisfaction. Chen et al. [25] investigated pharmaceutical logistics service quality with refined Kano’s model involving 104 respondents from medical institutions. Zhang et al. [26] studied on enhancing readers’ satisfaction model of electronic service quality in library based on LibQUAL+ and Kano model, and result shows that the model they built is feasible through the empirical analysis. However, for the research field of Eco-city, existing studies do not involve the Kano model to explore user (citizen who lives or works in the Eco-city) demands and the affection for the user satisfaction to date.

This paper aims to initially introduce the Kano model analysis method to the research field of user demands and satisfaction in terms of the Eco-city, intends to explore the relationship between the user demands and user satisfaction. Based on the Kano model, the user demands classification and importance for the Eco-city can be defined accordingly. The user demands analysis method of Eco-city proposed in this paper, can be used for other researchers worldwide to investigate and quantitively analyze user demands according to their local development situation and preference of Eco-city. The user demands analysis results obtained through this method, can benefit different stages of Eco-city. For the planning and design stage of Eco-city, user demands analysis can help avoid ‘empty city’ phenomenon after construction, so as to save huge amount of fund and time. Because the construction of Eco-cities is extremely complicated and time-consuming, the developing organization of Eco-city needs to allocate limited resources investing in facilities and services of more critical user demands. For the operation stage of Eco-city, user demands analysis can help conduct POE (Post Occupancy Evaluation) process, in order to reveal aspects that residents dissatisfied in terms of operation and management of Eco-city. The O&M organization of Eco-city will be able to undertake relevant renovations accordingly regarding facilities and services to maximize user satisfaction.

## 2. Materials

Since the Chinese national standard "Assessment Standard for green eco-district" (GB/T 51255–2017) [27] has been promulgated and implemented, the standard has strong authority, a large number of credits, and rich evaluation content. Therefore, the user demands library had been proposed based on the national standard.

Regarding the national standard "Assessment Standard for green eco-district", from the perspective of evaluation dimensions, it mainly includes government management dimension and user dimension. For example, the credit "4.1.1 urban planning should meet the urban and rural planning requirements of the region." It can be classified as the content of government management dimension. The credit "4.2.5 Public service facilities in residential areas has better convenience." can be classified as the content of user dimension, which reflects the actual needs and expectations of the people for an Eco-city. Therefore, this article had started from the user dimension, combed and categorized the content of the national standard "Assessment Standard for green eco-district" (GB/T 51255–2017), and initially summarized the user demands library including six major categories. The categories are land use, ecological environment, green building, energy utilization, green transportation and humanities, including 25 specific user demands, which are shown in Table 1.

**Table 1:** User demands library in Eco-city (Source: Authors, 2020).

Major categories	Serial no.	User demand item	Content overview
Land use	D1	Convenient public service facilities	There are kindergartens, nurseries, primary and secondary schools, pension service facilities, health service centers, commercial service facilities and other facilities around the residential area, which are convenient and accessible
	D2	Opening of public space	Public open space is set up in urban area for residents’ activities
	D3	Green space opening	The ecological land and urban green space with a certain scale and reasonable layout shall be maintained in the urban area
Ecological environment	D4	High greening rate	Three dimensional greening is implemented in the maintenance and management of all kinds of garden green space are good, and the greening coverage rate is high in the urban area
	D5	The rain water quick absorption	Implementation of sponge city construction, rainstorm without urban waterlogging
	D6	Avoid soil pollution	There is no soil pollution or the soil pollution control is completed and up to standard
	D7	High quality of domestic water	The quality of municipal domestic water reaches the national standard
	D8	Good outdoor air quality	The annual excellent days of air quality reached more than 240 days, and the average days of reaching the standard of PM2.5 reached more than 200 days
	D9	Good outdoor sound environment	The outdoor sound environmental quality of the area meets the national standard
	D10	Refuse classification	Classified collection, closed transportation and effective treatment of garbage
Green building	D11	New-built green building	Choose to buy green residential buildings or work in green office buildings
	D12	Green renovation of existing buildings	The residential buildings or office buildings that have been renovated to meet the requirements of national standard for green buildings
	D13	Green construction	The projects under construction meet the requirements of green construction and reduce the pollution and impact on the surrounding environment
	D14	Green operation	Green operation is adopted for residential buildings or office buildings, which can reduce operating costs and provide good building environment and high-quality services
Energy utilization	D15	Energy measurement	Charging for heating (cooling) consumption
	D16	Energy saving of municipal infrastructure	High efficiency lamps and light sources are used for road lighting, landscape lighting and traffic signal lights, and efficient equipment is used for municipal water supply and drainage pumps and related equipment
	D17	Reclaimed water use	Residential or office buildings have the conditions to utilize reclaimed water
Green transportation	D18	Public transport system	With high coverage of bus stops, bus lanes are set up, and the public transport system has humanized service facilities (such as guidance, barrier free access, sunshade, seats, etc.)
	D19	Bicycle transportation system	Urban bicycle lanes are continuous, with reasonable width to form a shady road, and complete road supporting facilities (such as guide signs, safety, rest, sanitation and other facilities)
	D20	Walking system	The walking system is continuous, combined with surrounding functions, environment, landscape and public space, with complete supporting facilities (such as lighting, guiding signs, safety, rest, sanitation and other facilities)
	D21	Charging facilities for new energy vehicles	Electric vehicle charging facilities should be built in residential buildings, public buildings and public parking lots
	D22	Parking spaces	The underground parking lot and public parking lot are provided with sufficient parking spaces, and the parking resources are efficiently utilized, and the parking information is timely and unobstructed
Humanity	D23	Elderly service facilities	The community service network for the aged is completed and there are enough beds for the aged
	D24	Street crossing facilities	Barrier free elevators or escalators are set up in overpasses and tunnels, voice signal lights for blind people crossing the street and button type signal lights for pedestrians at night are set at pedestrian crosswalks
	D25	Encourage green lifestyle	Formulate preferential measures to encourage residents to purchase energy-saving appliances and water-saving appliances

## 3. Methodology

China’s new urbanization process and the implementation of green development policies have jointly promoted the development of the Eco-cities. With the construction and operation of the Eco-city, user demands are constantly changing. According to the actual needs of users and feedback of problems encountered in the operation process, the Eco-city shall be continually improved and updated, and the functions and services need to meet user demands accordingly.

**Fig 1:**
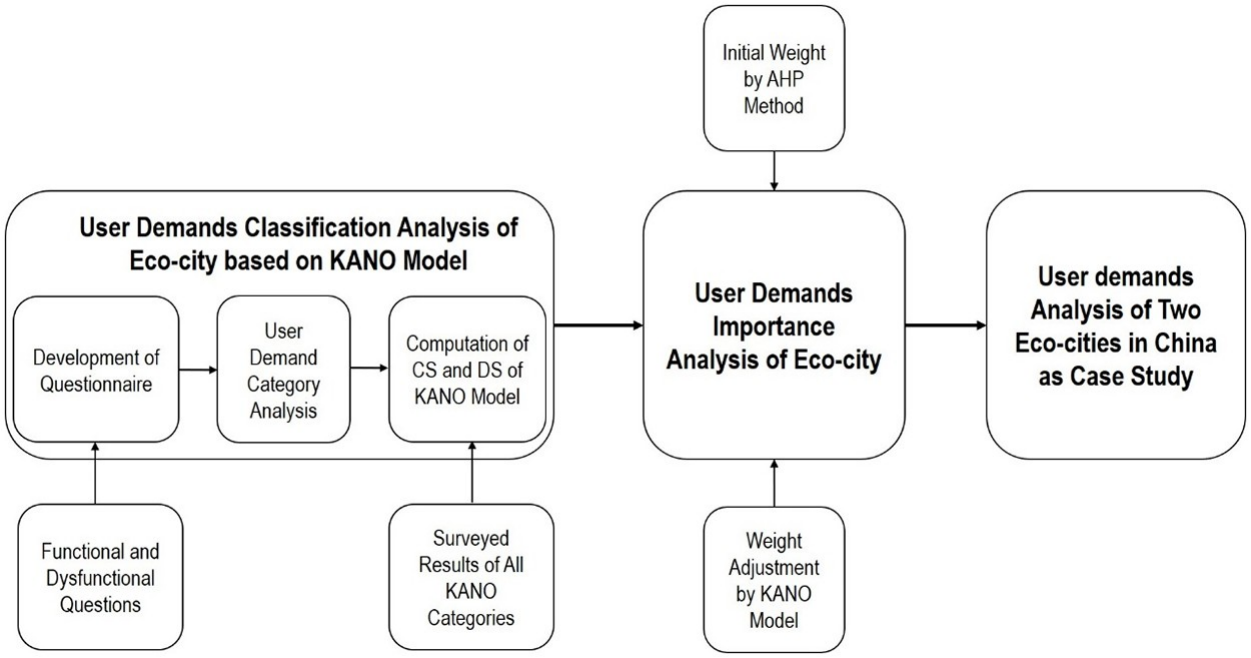
Illustration of the research methodology undertaken in this article. (Source: Authors, 2020).

In order to specifically understand the real demands of users in the operation of the Eco-city, and to improve the service quality, thereby enhancing the happiness index of users. The research process undertaken in this paper was as follows. Firstly, the user demands classification analysis for Eco-city based on Kano model was researched, including the questionnaire method and computation formula. Secondly the user demands importance analysis for Eco-city was studied. AHP method was adopted to obtain the initial weights of user demand items. Then weight adjustment was conducted based on the Kano demand category. Consequently, the final ranking of user demands importance was determined accordingly. At last, two Eco-cities in Tianjin and Chongqing city of China were selected to apply the methodology as case study. See Fig 1 for an illustration of the research process undertaken in this article.

### 3.1. User demands classification analysis of Eco-city based on Kano model

#### 3.1.1. Introduction of the Kano model

The Kano model was an important theoretical model which was initially proposed by Japanese quality management professor—Noriaki Kano in 1984 aiming to illustrate and identify quality attributes for the research of customer satisfaction [18]. Kano model provides a framework which could enable the elicitation of product requirements. It helps increase customer satisfaction if the elicited requirements are met [23]. Kano model not only could be used for requirement clarification in the early stage, but also for different stages in the service delivery lifecycle. Kano survey helps to add value by focusing efforts in service design, development and verification stages to encompass features on use case level, supported by early prototypes and conducted with real customers [24]. Compared with other models, the Kano Model does not assume the existence of a linear relationship between product/service performance and customer satisfaction. Kano noticed that customers’ requirements are not equivalent and that some requirements, in fact, are capable of generating more satisfaction than others. Moreover, customer satisfaction is not always proportional to the functionality of the good, which implies that higher quality does not necessarily lead to higher satisfaction for all product attributes or services requirements [28].

**Fig 2:**
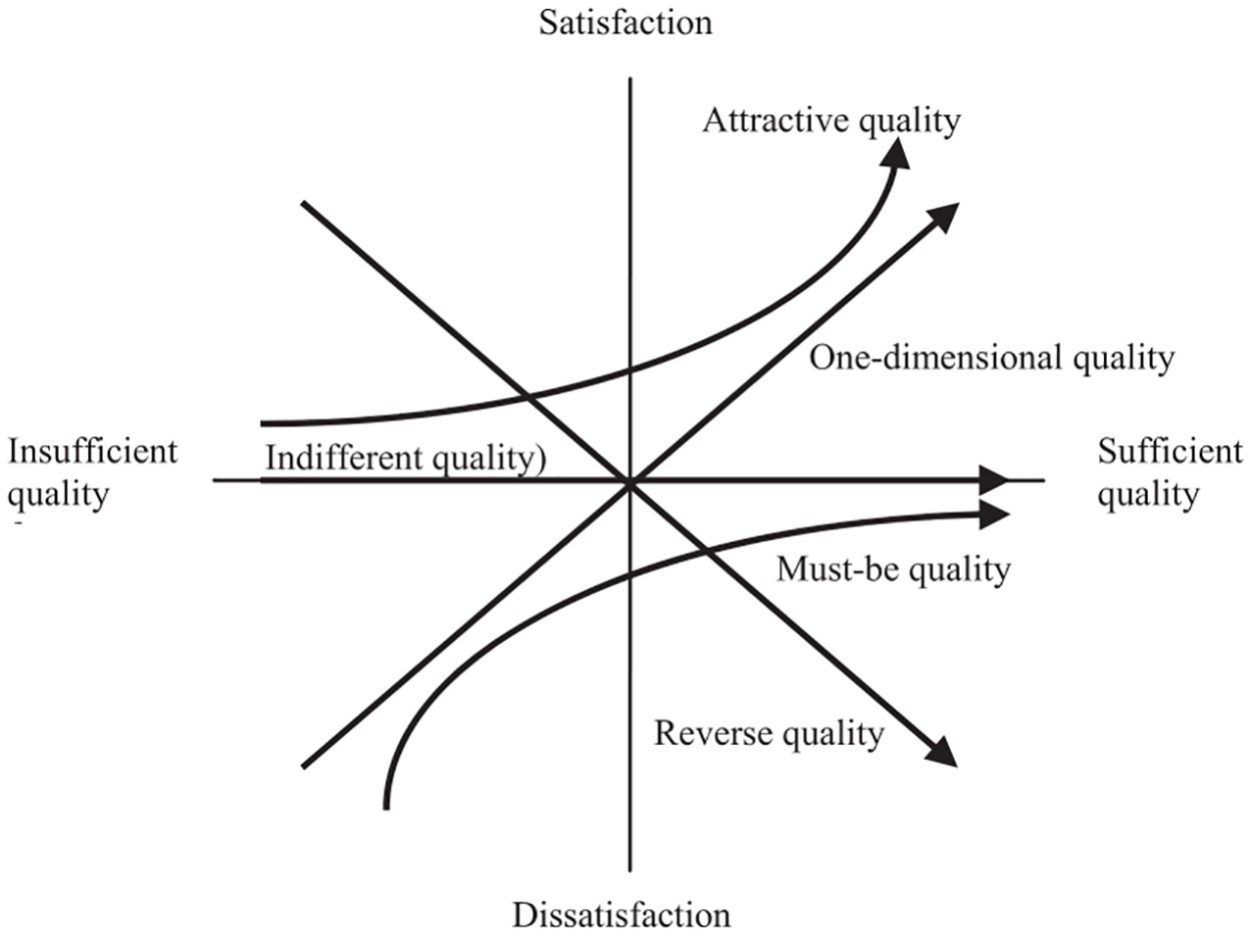
Relationship between product quality and user satisfaction of Kano model (Kano et al., 1984 [18]).

The customer satisfaction over specific quality of a product or service may vary with customer’s preference over the quality attribute as shown in Fig 2 [18]. The X -axis stands for the level of quality performance (from Insufficient to Sufficient) and the Y -axis represents the customer satisfaction level (from Dissatisfaction to Satisfaction). There are 5 quality attributes in Kano Model as follows [29]:

1. One-dimensional quality (O): Customer is satisfied when this quality attribute is sufficient and vice versa. Customer satisfaction level is in liner relation with quality attribute adequacy.
2. Attractive quality (A): Sufficiency of this quality attribute will lead to more satisfaction of customer, yet not cause dissatisfaction with the absence of this attribute.
3. Must-be quality (M): Customers tend to consider this quality attribute for granted. Which means improving this attribute will not result in more satisfaction, but the customer will become very dissatisfied if this quality attribute not provided.
4. Indifferent quality (I): Whether this quality attribute sufficient or not, will not affect customer satisfaction. The customer is not interested with the product or service quality.
5. Reverse quality (R): Customer does not desire this product attribute and also expects the reverse.

#### 3.1.2. The questionnaire of Kano model

A questionnaire can be constructed according to Kano model [30]. The questionnaire consists of questions about the functional requirements of a product. Each functional requirement consists of two inverse questions, one functional and one dysfunctional. For example, for the functional question, the customer (user) might be asked “If there are sufficient service facilities around the residential area, which are very convenient and accessible, how do you feel?”. For the dysfunctional question, the customer (user) might be asked “If there are insufficient service facilities around the residential area, which are not convenient and accessible, how do you feel?”. Each functional and dysfunctional question has five possible answers, (1) I like it that way; (2) It must be that way; (3) I am neutral; (4) I can live with it that way; (5) I dislike it that way.

#### 3.1.3. User demand category analysis of Kano model

The form shown in Table 2 can be utilized to evaluate the quality attribute category of demand by each customer (user), including One-dimensional (O), Attractive (A), Must-be (M), Indifferent (I), and Reverse (R). “Q” represents unusable response which will be eliminated. Then all the Kano categories for each demand item of all customers (users) are summarized to determine the final Kano category for each demand item, with adopting the principle of relatively majority [31].

**Table 2:** Kano evaluation form for quality attribute category (Source: Authors, 2020).

Functional question	Dysfunctional question				
	Like	Must-be	Neutral	Live with	Dislike
Like	Q	A	A	A	O
Must-be	R	I	I	I	M
Neutral	R	I	I	I	M
Live with	R	I	I	I	M
Dislike	R	R	R	R	Q

#### 3.1.4. Computation of CS and DS of Kano model

After collecting all the responses from customers (users) towards each demand item in terms of the Kano category (One-dimensional, Attractive, Must-be, etc.), it is possible to calculate two coefficients, namely customer satisfaction (CS) and customer dissatisfaction (DS) [30]. The customer satisfaction coefficient has a value between 0 and 1 (values close to 1 represent great satisfaction while values close to 0 indicate low satisfaction). The customer dissatisfaction coefficient has a value between -1 and 0 (values close to -1 represent great dissatisfaction while values close to 0 indicate low dissatisfaction). The calculated equations are as follows:

$$CS=\frac{A_{i}+O_{i}}{A_{i}+O_{i}+M_{i}+I_{i}}$$

$$DS=\frac{M_{i}+O_{i}}{A_{i}+O_{i}+M_{i}+I_{i}}.(-1)$$

When *A*_*i*_, *O*_*i*_, *M*_*i*_, *I*_*i*_ stands for the number of Attractive, One-dimensional, Must-be, and Indifferent attribute respectively of demand item i.

### 3.2. User demands importance analysis of Eco-city based on Kano model

#### 3.2.1. The questionnaire of user demands importance

The user demands survey questionnaire of Kano model can carry out qualitative analysis of classification for each demand. However, to understand the comprehensive importance of each user demand in detail, further user surveys are needed. Therefore, Likert five-point scale method was used for the importance survey questionnaires, setting "very unimportant", "relatively unimportant", "generally important", “relatively important” and "very important" for each user demand item [32]. These five levels correspond to scores of 1, 2, 3, 4, and 5 respectively. The importance questionnaire sample form of user demand in Eco-city indicates as follows in Table 3.

**Table 3:** The importance questionnaire sample form of user demand in Eco-city (Source: Authors, 2020).

Serial number	Content overview	Very unimportant	Relatively unimportant	Generally important	Relatively important	Very important
D1	There are kindergartens, nurseries, primary and secondary schools, pension service facilities, health service centers, commercial service facilities and other facilities around the residential area, which are convenient and accessible					

#### 3.2.2. Determination of initial weight

The most mature AHP (Analytic Hierarchy Process) method [33,34] in the industry was used to determine the initial weights of the importance of 25 user demands in the Eco-city. This method can summarize different factors related to decision-making, and perform qualitative and quantitative evaluation [35,36]. When using this method for calculation, the judgment matrix needs to be constructed firstly. The way to construct the judgment matrix is: calculate the average score of each analysis item, and then divide the average score for two items successively to form judgment matrix. For the user demands importance analysis of eco-city, 25-order judgment matrix was constructed (because 25 user demand items in the survey). After summarizing the results of importance questionnaire for eco-city, the average score for each demand item was calculated, and the judgment matrix was shown in Table 4 (as the whole matrix was too large, only 10-order matrix shown for demonstration).

**Table 4:** Judgment matrix for user demand items of Eco-city (Source: Authors, 2020).

Average Score	No. of demand item	D-1	D-2	D-3	D-4	D-5	D-6	D-7	D-8	D-9	D-10	…
4.162	D-1	1	1.027	1.015	1.013	0.998	0.991	0.973	0.984	0.989	1	…
4.054	D-2	0.974	1	0.989	0.987	0.972	0.965	0.948	0.958	0.963	0.974	…
4.1	D-3	0.985	1.011	1	0.998	0.983	0.976	0.959	0.969	0.974	0.985	…
4.108	D-4	0.987	1.013	1.002	1	0.985	0.978	0.96	0.971	0.976	0.987	…
4.169	D-5	1.002	1.028	1.017	1.015	1	0.993	0.975	0.985	0.991	1.002	…
4.2	D-6	1.009	1.036	1.024	1.022	1.007	1	0.982	0.993	0.998	1.009	…
4.277	D-7	1.028	1.055	1.043	1.041	1.026	1.018	1	1.011	1.016	1.028	…
4.231	D-8	1.017	1.044	1.032	1.03	1.015	1.007	0.989	1	1.005	1.017	…
4.208	D-9	1.011	1.038	1.026	1.024	1.009	1.002	0.984	0.995	1	1.011	…
4.162	D-10	1	1.027	1.015	1.013	0.998	0.991	0.973	0.984	0.989	1	…
…	…	…	…	…	…	…	…	…	…	…	…	…

The judgment matrix analysis used to get the eigenvector and initial weight of each user demand item. Then a consistency test should be conducted. The maximum eigenvalue according to the vectors of matrix needs to be calculated, and the maximum eigenvalue utilized to get the CI (consistency index = (maximum eigenvalue–n)/(n-1)). RI (Random Consistency Index) can be referred to Table 5 according to the order of matrix (For example, the RI of 25-order matrix is 1.6556).

**Table 5:** Random consistency index (Source: Saaty, 2008).

Order n	3	4	5	6	7	8	9	10	11	12	13	14	15	16
RI	0.52	0.89	1.12	1.26	1.36	1.41	1.46	1.49	1.52	1.54	1.56	1.58	1.59	1.5943
Order n	17	18	19	20	21	22	23	24	25	26	27	28	29	30
RI	1.6064	1.6133	1.6207	1.6292	1.6358	1.6403	1.6462	1.6497	1.6556	1.6587	1.6631	1.667	1.6693	1.6724

Finally, a consistency test is needed to calculate the CR (consistency ratio = CI/RI). Under normal circumstances, the smaller the value obtained by CR, the better, because this represents better consistency of the key calculation matrix and the normalization. Generally speaking, the value of the calculated correlation matrix should not be greater than 0.1 [34].

#### 3.2.3. Weight adjustment of user demand items based on Kano model

Initially, the user’s awareness is the only way to determine the initial importance, and it is difficult for users to notice the impact of their own needs on satisfaction, which leads to the great limitations of this evaluation method. Through the analysis of user feedback after the return visit, it is found that most users will emphasize their essential functions when using them, or hope that the product can increase the actual functions they need, but almost no users express their needs for attractive attributes. Attractive attributes can bring unimaginable parts to users in the subconscious to increase their satisfaction, which is an aspect that users often overlook when using products [37].

Therefore, in the final weight analysis, the initial weight of the user demand item cannot be directly used to analyze the importance of the user demand item. It is also necessary to consider whether the demand item is helpful for improving user satisfaction. This paper adjusted the initial weight of each demand item based on the theory of the Kano model, so as to obtain the final weight of user demand in the Eco-city [38]. The adjustment formula is as follows:

$$w_{i}^{\prime }=\frac{w_{i}\times m_{i}}{\sum_{i=1}^{n}w_{i}\times m_{i}}(i=1,2,\ldots ,n)$$

Where *w*_*i*_—Initial weight of user demand item i; $w_{i}^{'}$—Final weight of user demand item i; *m*_*i*_—Kano classification adjustment coefficient of user demand item i;

When the user demand item classified to Attractive attribute, *m*>1;

When the user demand item classified to One-dimensional attribute, *m* = 1;

When the user demand item classified to Must-be attribute, 0<*m*<l.

#### 3.2.4. Ranking of user demands importance

Because the final weight of demand items not only reflects the user’s evaluation of the importance of each demand item, but also reflects the impact of each demand item on improving user satisfaction, so the comprehensive importance of user demand items can be ranked according to the final weight. In the operation and management stage of the Eco-city, the operation and management works are extremely complicated. The Eco-city operation manager can refer to the importance of the user demands, and put more resources and energy on the most important demand items. Which can effectively improve user satisfaction and reputation, and truly achieve the goal of "people-oriented".

### 3.3. Case study of user demands analysis of Eco-city based on Kano model

#### 3.3.1 Basic information of eco-cities selected for case study

Sino-Singapore Tianjin Eco-city and Chongqing Yuelai Eco-city of China were selected to apply the methodology of user demands analysis as a case study. The specifications of the two Eco-cities are described in Table 6.

**Table 6:** The specifications of the two Eco-cities for case study (Source: Authors, 2020).

Specification	Sino-Singapore Tianjin Eco-city	Chongqing Yuelai Eco-city
Location	Tianjin city, China	Chongqing City, China
Region	Northern China	Southwestern China
Climate zone	Cold zone	Hot-summer and cold-winter zone
Zone area	31.23 km^2^	18 km^2^
Population	Approximately 100,000	Approximately 500,000

#### 3.3.2 Survey questionnaire preparation

After determining the user demands items that need to be investigated in section 2, this paper designed a user survey questionnaire according to the requirements of the Kano model. The survey was mainly divided into three parts. The first part was to conduct a basic background survey of the users in the Eco-city. This part sets a total of eight questions, such as gender, age, education, occupation, location of work and living, and the awareness of the concepts of Eco-city. The second part was to ask functional and dysfunctional questions for the demand items of the Eco-city mentioned in this article, so as to understand the attitudes and expectations of the surveyed users for each user demand item. The third part is the survey of the importance of user demands in the Eco-city.

#### 3.3.3 Distribution and collection of survey questionnaires

The user demands questionnaire survey was conducted from September 1^st^ to October 31^th^, 2019. Sampling size (n) was computed which should be more than 123 by using the formula [39] below.

$$n=\frac{Z^{2}NPQ}{\left(ND^{2})+(Z^{2}PQ\right)}$$

Where n = Sampling size, N = Population size, Z = confidence coefficient (1.96 for % 95), P = probability of measurement in population (0.8), Q = 1- P (0.2) and D = sampling error (0.1).

The online questionnaires via a website (https://www.wjx.cn) were distributed to users of the selected Eco-cities for case study (people who work or live in Sino-Singapore Tianjin Eco-city and Chongqing Yuelai Eco-city of China). The survey participants were able to finish the online questionnaires generated by the website either by computer or mobile phone. All the data was collected anonymously. A statement “All data collected will only be used for research of user demands analysis in terms of eco-city, submission of the questionnaire means that you give consent to the data collection. Thank you!” was included in the questionnaire. A total of 156 questionnaires were returned in this survey which could fulfil the sampling size computed (more than 123).

## 4. Results

### 4.1. Distribution and collection of survey questionnaires

A total of 156 questionnaires were returned for this survey in the selected eco-cities. Among the 156 collected questionnaires, 26 invalid questionnaires were excluded, because they both selected the “Like” or “Dislike” for functional question and dysfunctional question corresponding to “Q” according to Table 2 in this paper. According to the collected 130 valid questionnaires, the basic information summary of the surveyed users is shown in Table 7.

**Table 7:** Basic information summary of the surveyed users in the two Eco-cities (Source: Authors, 2020).

Item	Subtype	Number of People	Percentage
Gender	Male	68	52.31%
	Female	62	47.69%
Age	18 or less	1	0.77%
	18~25	14	10.77%
	26~30	22	16.92%
	31~40	67	51.54%
	41~50	22	16.92%
	51~60	4	3.08%
	Over 60	0	0%
Living area	Tianjin Sino-Singapore Eco-city	62	47.69%
	Chongqing Yuelai Eco-city	56	43.08%
	Other	12	9.23%
Working area	Tianjin Sino-Singapore Eco-city	76	58.46%
	Chongqing Yuelai Eco-city	38	29.23%
	Other	16	12.31%
Awareness of concept of the Eco-city	Unfamiliar	18	13.85%
	Generally familiar	56	43.08%
	Relatively familiar	33	25.38%
	Very familiar	23	17.69%

Reliability and validity tests of collected valid survey questionnaires were conducted before further analysis of Kano category and importance of user demands. This process requires the use of SPSS software.

The final results of the reliability and validity of the Kano questionnaire and importance questionnaire are shown in Tables 8 and 9. The Cronbach’s α coefficient of the responses to functional questions, dysfunctional questions and importance questions are 0.948, 0.986 and 0.993 respectively. Obviously, these three data are very close to 1, which shows that the data obtained through the Kano questionnaire and importance questionnaire with high quality of reliability [40]. The KMO values of the responses to functional questions, dysfunctional questions and importance question are 0.819, 0.951 and 0.960 respectively which are all more than 0.8, which demonstrates good validity [41].

**Table 8:** Reliability and validity tests results for Kano questionnaire survey (Source: Authors, 2020).

Type of test		Responses to functional questions	Responses to dysfunctional questions
**Reliability**	Cronbach’s α coefficient	0.948	0.986
**Validity**	KMO value	0.819	0.951
	Bartlett’s test value	3502.217	5833.223
	Sig value	0.000	0.000
	Accumulated variance contribution rate	80.938%	80.777%

**Table 9:** Reliability and validity tests results for importance questionnaire survey (Source: Authors, 2020).

Type of test		Responses to importance questions
**Reliability**	Cronbach’s α coefficient	0.993
**Validity**	KMO value	0.960
	Bartlett’s test value	6648.845
	Sig value	0.000
	Accumulated variance contribution rate	86.159%

### 4.2. Analysis of Kano category results of user demands

The answer data of each surveyed user was sorted and analyzed correspond to the Kano evaluation form, to determine the Kano category for each demand item for the user. Then all the Kano categories for each demand item of all users were summarized to determine the final Kano category for each demand item, with adopting the principle of relatively majority. According to the Kano category distribution of each demand item, the CS value (the Customer satisfaction coefficient) and the DS value (the Customer dissatisfaction coefficient) were calculated for each demand item, to obtain the impact of each demand item on the level of user satisfaction. The Kano category distribution of each user demand item in the Eco-city, and the calculated CS value and DS value for each demand item are summarized in Table 10.

**Table 10:** Kano category and CS & DS value of each user demand item (Source: Authors, 2020).

Serial No.	User demand item	A	O	M	I	R	Kano category	CS value	DS value
D-1	Convenient public service facilities	14	34	48	21	1	M	0.407	-0.695
D-2	Opening of public space	12	77	8	22	0	O	0.748	-0.714
D-3	Green space opening	10	85	6	18	1	O	0.792	-0.758
D-4	High greening rate	11	80	8	21	0	O	0.758	-0.733
D-5	The rain water quick absorption	5	85	14	16	1	O	0.744	-0.818
D-6	Avoid soil pollution	3	89	18	13	1	O	0.742	-0.863
D-7	High quality of domestic water	3	90	14	12	2	O	0.769	-0.860
D-8	Good outdoor air quality	10	89	7	16	0	O	0.811	-0.787
D-9	Good outdoor sound environment	10	83	15	12	0	O	0.775	-0.817
D-10	Refuse classification	7	83	16	14	1	O	0.744	-0.818
D-11	New-built green building	14	67	8	32	0	O	0.669	-0.620
D-12	Green renovation of existing buildings	16	68	7	29	1	O	0.694	-0.620
D-13	Green construction	6	39	59	16	0	M	0.375	-0.817
D-14	Green operation	58	33	13	16	0	A	0.758	-0.383
D-15	Energy measurement	13	71	12	24	2	O	0.689	-0.680
D-16	Energy saving of municipal infrastructure	12	77	13	20	0	O	0.730	-0.738
D-17	Reclaimed water use	9	66	7	35	3	O	0.625	-0.608
D-18	Public transport system	11	76	12	21	0	O	0.725	-0.733
D-19	Bicycle transportation system	11	78	11	17	3	O	0.742	-0.742
D-20	Walking system	14	79	10	16	1	O	0.775	-0.742
D-21	Charging facilities for new energy vehicles	11	76	6	25	4	O	0.713	-0.672
D-22	Parking spaces	13	17	73	16	3	M	0.246	-0.738
D-23	Elderly service facilities	9	75	7	29	1	O	0.694	-0.678
D-24	Street crossing facilities	11	78	9	22	1	O	0.736	-0.719
D-25	Encourage green lifestyle	13	73	5	28	1	O	0.717	-0.650

According to the analysis results of the Kano category of each demand item in Table 10, the user demands of the Eco-city were mainly including three categories: Attractive demand (A), One-dimensional demand (O), and Must-be demand (M). The Kano categories of user demands are summarized in Table 11.

**Table 11:** Kano category summary of user demand item in Eco-city (Source: Authors, 2020).

Kano category	User demand item in Eco-city
Attractive demand (A)	Green operation
One-dimensional demand (O)	Opening of public space, Green space opening, High greening rate, The rain water quick absorption, Avoid soil pollution, High quality of domestic water, Good outdoor air quality, Good outdoor sound environment, Refuse classification, New-built green building, Green renovation of existing buildings, Energy measurement, Energy saving of municipal infrastructure, Reclaimed water use, Public transport system, Bicycle transportation system, Walking system, Charging facilities for new energy vehicles, Elderly service facilities, Street crossing facilities, Encourage green lifestyle
Must-be demand (M)	Convenient public service facilities, Green construction, Parking spaces

There was only one user demand item that belongs to the Kano category of Attractive demand, which was D-14 Green operation, and the demand item belongs to major category “Green building”. It can be seen from Table 10 that there was a large gap between the absolute value of the CS value (the influence of the demand item on improving user satisfaction) and the absolute value of the DS value (the influence of the demand item on reducing user satisfaction). The absolute value of CS value was above 0.5, but the absolute value of DS was small, which means that when this demand item has a high degree of satisfaction, it can significantly enhance user satisfaction, but with a low degree, it will not bring users strong dissatisfaction. It is widely known that green buildings are the basic units and cells of an Eco-city. Studies have shown that 80% to 90% of a person’s life spent inside buildings. If the buildings in the Eco-city cannot be truly green, then the overall Eco-city also difficult to truly establish a reputation. Therefore, the improvement of Attractive demand (D-14 Green operation) can make the overall service level of the Eco-city more perfect.

There were three user demand items belonging to the Kano category of Must-be demand, with the serial number D-1, D-13, and D-22. The major demand categories involved were “Land use”, “Green building”, and “Green transportation”. It can be seen from Table 10 that the absolute value of DS of the three demand items was relatively high, while the absolute value of CS was relatively low. When such demand items are not available or have a low degree of supply, it will greatly reduce the user satisfaction level. Therefore, the operation and management organization of Eco-city needs to ensure that the Must-be demands are met for users. From the perspective of the major demand categories, the operation and management organization of Eco-city should continuously improve the supporting service facilities around the residential area (such as kindergartens, nurseries, primary and secondary schools, elderly service facilities, health service centers, commercial service facilities, etc.) to ensure that basic service requirements of the residents in the Eco-city are met, and service levels constantly improved. In addition, because the construction of an Eco-city is not achieved overnight, the construction project basically in the state of being constructed and put into use at the same time, and the goal of realization for green construction must be achieved. With the improvement of people’s living standards, motor vehicles have become a necessity for families, and as a harmonious and livable Eco-city, "difficult parking" should not become a problem that plagues residents’ daily life.

There are 21 user demand items that belong to the Kano category of One-dimensional demand, and the major demand categories cover “Land use”, “Green building”, “Energy utilization”, “Green transportation” and “Humanities”. The absolute value of CS value and DS value for the 21 demand items were all relatively high above 0.5, and the difference between CS and DS was very small, which has a greater impact on user satisfaction and dissatisfaction, directly proportional to user satisfaction. One-dimensional demand items are the key functional requirements that the Eco-city needs to pay attention to. Therefore, the operation and management organization of Eco-city needs to pay attention to the maintenance and improvement of such demand items, and try their best to meet such needs, so as to maximize the user satisfaction and reduce user dissatisfaction. The operation and management organization of Eco-city needs to pay attention to the expectations and needs of users from multiple perspectives of land use, green buildings, energy utilization, green transportation, and humanities. However, the operation and management of Eco-city are extremely complicated, and the organization needs to allocate limited resources investing in more critical user demands.

### 4.3. Analysis of the importance of user demands

#### 4.3.1. Analysis of initial weight results for user demand item

In this paper, AHP analysis method was used to calculate the initial weight of the importance of each user demand item of the Eco-city, and the calculation tool was SPSS software. 25-order judgment matrix was constructed, the eigenvector and initial weight value for each user demand item calculated by AHP method are Shown in Table 12. And the calculated results have passed the consistency test of AHP method (CI = 0.000, RI = 1.656, CR < 0.1).

**Table 12:** Initial weight results for user demand items calculated by AHP method (Source: Authors, 2020).

Serial No. of user demand item	Eigenvector	Initial weight
D-1	1.024	4.097%
D-2	0.998	3.991%
D-3	1.009	4.037%
D-4	1.011	4.044%
D-5	1.026	4.105%
D-6	1.034	4.135%
D-7	1.053	4.211%
D-8	1.041	4.165%
D-9	1.036	4.143%
D-10	1.024	4.097%
D-11	0.949	3.794%
D-12	0.935	3.741%
D-13	1.003	4.014%
D-14	0.996	3.984%
D-15	0.966	3.862%
D-16	0.981	3.923%
D-17	0.937	3.749%
D-18	1.009	4.037%
D-19	1.002	4.006%
D-20	1.017	4.067%
D-21	0.994	3.976%
D-22	1.017	4.067%
D-23	0.973	3.893%
D-24	0.988	3.953%
D-25	0.977	3.908%

### 4.3.2. Analysis of adjustment weight results for user demand item

Weight adjustment analysis were conducted for each user demand item in the Eco-city combining with the classification of the Kano categories, which mainly involving Attractive demands (A), One-dimensional demands (O), and Must-be demands (M) in this paper. When a user demand item belongs to the category of One-dimensional demand, the user’s satisfaction with such a demand item is directly proportional to its degree of availability. Therefore, the value of adjustment coefficient (m) usually equals to 1.0 [18]. When the user demand item belongs to the category of the Attractive demand, compared with the One-dimensional demand item, it is an unexpected demand of the user, which is more helpful for improvement of user satisfaction. Hence the value of m is usually greater than 1.0. When the user demand item belongs to the category of the Must-be demand, the user considers that this kind of demand item is the prerequisite demand item. When such demand is not fulfilled, the user will be very dissatisfied. However, even if such demand items have a high degree of availability or exceed user expectations, the degree of user satisfaction will not improve significantly. Such demand items are not very helpful in improving user satisfaction. Therefore, the value of m for Must-be demand items is often selected from 0 to 1.0.

For the user demand items of the Attractive demand and Must-be demand, the value of the adjustment coefficient m has not been fixed in the existing research. By referring to the relevant literatures [42], the value of adjustment coefficient m for the Attractive demand was assigned as 1.1 in this paper, and the value of adjustment coefficient m for the Must-be demand was assigned as 0.9. Then the final weight of each demand item was calculated with initial weight and adjustment coefficient according to the formula in section 3.2.3. The values of the adjustment coefficient m and final weights for the 25 user demand items are shown in Table 13.

**Table 13:** The values of the adjustment coefficient (m) and final weight for the user demand items (Source: Authors, 2020).

Serial No.	User demand item	Kano category	Adjustment coefficient (m)	Final weight
D-1	Convenient public service facilities	M	0.9	3.718%
D-2	Opening of public space	O	1	4.024%
D-3	Green space opening	O	1	4.070%
D-4	High greening rate	O	1	4.077%
D-5	The rain water quick absorption	O	1	4.139%
D-6	Avoid soil pollution	O	1	4.169%
D-7	High quality of domestic water	O	1	4.246%
D-8	Good outdoor air quality	O	1	4.199%
D-9	Good outdoor sound environment	O	1	4.177%
D-10	Refuse classification	O	1	4.131%
D-11	New-built green building	O	1	3.825%
D-12	Green renovation of existing buildings	O	1	3.772%
D-13	Green construction	M	0.9	3.642%
D-14	Green operation	A	1.1	4.419%
D-15	Energy measurement	O	1	3.894%
D-16	Energy saving of municipal infrastructure	O	1	3.955%
D-17	Reclaimed water use	O	1	3.780%
D-18	Public transport system	O	1	4.070%
D-19	Bicycle transportation system	O	1	4.039%
D-20	Walking system	O	1	4.101%
D-21	Charging facilities for new energy vehicles	O	1	4.009%
D-22	Parking spaces	M	0.9	3.691%
D-23	Elderly service facilities	O	1	3.925%
D-24	Street crossing facilities	O	1	3.986%
D-25	Encourage green lifestyle	O	1	3.940%

#### 4.3.3. Importance ranking result analysis for user demand item

The 25 user demands items were sorted in descending order according to their final weights, the overall importance ranking of user demand items in the Eco-city are shown in Table 14.

**Table 14:** The overall importance ranking of user demand items (Source: Authors, 2020).

Serial No.	User demand item	Major demand category	Kano category	Final weight	Importance ranking
D-14	Green operation	Green building	A	4.42%	1
D-7	High quality of domestic water	Ecological environment	O	4.25%	2
D-8	Good outdoor air quality	Ecological environment	O	4.20%	3
D-9	Good outdoor sound environment	Ecological environment	O	4.18%	4
D-6	Avoid soil pollution	Ecological environment	O	4.17%	5
D-5	The rain water quick absorption	Ecological environment	O	4.14%	6
D-10	Refuse classification	Ecological environment	O	4.13%	7
D-20	Walking system	Green transportation	O	4.10%	8
D-4	High greening rate	Ecological environment	O	4.08%	9
D-3	Green space opening	Land use	O	4.07%	10
D-18	Public transport system	Green transportation	O	4.07%	11
D-19	Bicycle transportation system	Green transportation	O	4.04%	12
D-2	Opening of public space	Land use	O	4.02%	13
D-21	Charging facilities for new energy vehicles	Green transportation	O	4.01%	14
D-24	Street crossing facilities	Humanity	O	3.99%	15
D-16	Energy saving of municipal infrastructure	Energy utilization	O	3.96%	16
D-25	Encourage green lifestyle	Humanity	O	3.94%	17
D-23	Elderly service facilities	Humanity	O	3.93%	18
D-15	Energy measurement	Energy utilization	O	3.89%	19
D-11	New-built green building	Green building	O	3.83%	20
D-17	Reclaimed water use	Energy utilization	O	3.78%	21
D-12	Green renovation of existing buildings	Green building	O	3.77%	22
D-1	Convenient public service facilities	Land use	M	3.72%	23
D-22	Parking spaces	Green transportation	M	3.69%	24
D-13	Green construction	Green building	M	3.64%	25

Compared with Kano category classification analysis for the 25 user demand items, it is found that the Attractive demand and the One-dimensional demand ranked higher than the Must-be demand. These two types of demand items will have a relatively large impact on improving user satisfaction, indicating that the demand items that users are more willing to accept, and also the more important demand items. The importance ranking results reflect not only the importance of each demand item, but also the degree of influence on the improvement of user satisfaction.

From the perspective of the major demand categories of user demands library in Eco-city mentioned in section 2 of this paper, as for the top 10 demand items, "Green operation", "Walking system" and "Green space opening" belong to the major demand categories of "Green building", "Green transportation" and "land use" respectively. The other demand items are all demand items in the major demand category of "Ecological environment", which means that the users of the Eco-city pay more attention to the ecological environment. Ecological environment is an important field and the carrier of key indicators for the operation monitoring of the Eco-city.

The major categories of demand items ranked 11–25 are basically sorted into "Green transportation", "Humanities", and "Energy utilization" according to their importance. It can be seen that with the gradual improvement of people’s living standards, users of Eco-cities have relatively low attention to the effective use of resources. Compared with them, they pay more attention to the convenient transportation and humanized functions of Eco-cities.

## 5. Discussion

This study mainly focused on the analysis of user demands of Eco-city based on the Kano Model. The methodology can be used for other researchers to investigate and quantitively analyze user demands according to local development situation and preference of Eco-city. However, there are still some limitations of results in this paper.

Firstly, due to time constraints, the questionnaires were mainly distributed to the citizens who live or work in Sino-Singapore Tianjin Eco-city and Chongqing Yuelai Eco-city of China. As the development and construction of Eco-cites in China, there were several Chinese Eco-cites entering the operation stage successively. Extensive surveys need to be conducted in the future for different types of Eco-cities in different regions of China, to explore the similarity and difference of user demands in the Eco-cities of China. Secondly, based on the user demands importance ranking results in this study, the green operation of green buildings and ecological environment are the most important demands of the two surveyed Eco-cites. The results in this paper in terms of user demands importance will be delivered to the management organization of Tianjin and Chongqing Eco-cites of China. They will be able to improve the facilities and services according to the results, and user satisfaction survey will be conducted to further validate the analysis results proposed in this paper.

## 6. Conclusions

This paper initially introduced the Kano model analysis method to the research field of user demands in Eco-city, to explore the relationship between the user demand and user satisfaction. Based on the user demand analysis of two Eco-cities as case study, the following conclusions can be drawn:

1. The user demands analysis method based on Kano model for Eco-city proposed in this paper has been validated to be feasible. Comparing with the existing literatures in terms of user demands research for Eco-city, the user demands analysis method based on Kano model of this paper, is able to reveal the influence degree of user satisfaction towards the facilities and services provided in the Eco-city. Referring to the importance ranking adjusted by Kano model of user demands, the operation and management organization of Eco-city will be able to allocate limited resources investing in facilities and services for more critical user demands to maximize the user satisfactions, which will truly achieve the goal of "people-oriented" for Eco-city.
2. Based on the case study for two Eco-cities in China, it can be found that: 1) Compared with Kano category classification analysis for the 25 user demand items, the Attractive demand and the One-dimensional demand ranked higher than the Must-be demand. These two Kano categories of demand items will have a relatively large impact on improving user satisfaction, indicating that the demand items which users are more willing to accept, and also the more important demand items. 2) From the perspective of the major demand categories of user demands library in Eco-city mentioned in this paper, the top 10 demand items obtained, "Green operation", "Walking system" and "Green space opening" belong to the major demand categories of "Green building", "Green transportation" and "land use" respectively. The other demand items are all demand items in the major demand category of "Ecological environment", which means that the users of the two Eco-cities as case study pay more attention to the ecological environment.

Although this study conducted user demands analysis selecting two Eco-cities in China as a case study. The method of analysis of user demands classification and importance ranking for Eco-city can be applied to other areas around the world. Firstly, the researcher should select the most relevant local standards and regulations in terms of Eco-city to establish a user demands library in Eco-city. Secondly, based on the user demands library in Eco-city, the questionnaires of Kano model and importance survey need to be distributed to the local citizens who live or work in the sample Eco-city. Thirdly, the quantitative analysis method of Kano model, the initial weight and weight adjustment analysis method for user demands can be introduced to figure out the final importance ranking for the user demands of local citizens in Eco-city.

## Supporting information

S1 File (DOCX)
