# Supplementary material for: User demands analysis of Eco-city based on the Kano model—An application to China case study
Source: PLoS One. 2021 Mar 10;16(3):e0248187. doi: 10.1371/journal.pone.0248187 (PMC7946302; doi:10.1371/journal.pone.0248187)
Supplement: S1 File — (DOCX) [file pone.0248187.s001.docx]

**绿色生态城区用户需求调查问卷**

十分感谢您可以配合我们来填写此次问卷。通过本问卷，主要想了解一下您对于居住/工作的城区在绿色生态方面的实际需求，以及对其未来发展有何建议。您在填写此次问卷的时候，并不需要备注自己的姓名，其中所有的题目都属于单选类型。同时，此次问卷的内容仅仅用来提供学术上的研究，您的配合给了我们极大的帮助与鼓励，再次感谢您对我们的支持。

一、您的背景信息

1. 性别： [单选题] *

| ○男 | ○女 |  |  |  |  |  |  |
| --- | --- | --- | --- | --- | --- | --- | --- |

2. 您的年龄段： [单选题] *

| ○18岁以下 | ○18~25 | ○26~30 | ○31~40 | ○41~50 | ○51~60 | ○60以上 |
| --- | --- | --- | --- | --- | --- | --- |

3. 您目前从事的职业： [单选题] *

| ○全日制学生 |
| --- |
| ○生产人员 |
| ○销售人员 |
| ○市场/公关人员 |
| ○客服人员 |
| ○行政/后勤人员 |
| ○人力资源 |
| ○财务/审计人员 |
| ○文职/办事人员 |
| ○技术/研发人员 |
| ○管理人员 |
| ○教师 |
| ○顾问/咨询 |
| ○专业人士(如会计师、律师、建筑师、医护人员、记者等) |
| ○其他 |

4. 最高学历： [单选题] *

| ○高中/中专及以下 |
| --- |
| ○大专 |
| ○本科 |
| ○硕士及以上 |

5. 居住的地区为： [单选题] *

| ○中新天津生态城片区 |
| --- |
| ○重庆两江新区悦来片区 |
| ○其他地区 _________________ |

6. 工作的地区为： [单选题] *

| ○中新天津生态城片区 |
| --- |
| ○重庆两江新区悦来片区 |
| ○其他地区 _________________ |

7. 您是否了解绿色建筑的概念： [单选题] *

| ○不了解 |
| --- |
| ○一般了解 |
| ○比较了解 |
| ○非常了解 |

8. 您是否了解绿色生态城区的概念： [单选题] *

| ○不了解 |
| --- |
| ○一般了解 |
| ○比较了解 |
| ○非常了解 |

二、绿色生态城区需求调查
请您依据您的实际感受来选择，每一项需求包含正反两方面的描述。
每个问题涵盖五个答案：喜欢、理应如此、无所谓、可以忍受、不喜欢。请根据您真实感受的不同程度进行选择。
**请您注意对某一项需求的正反两方面描述的选择答案，不能同时选择“喜欢”或者同时选择“不喜欢”，否则会被判为无效问卷。**

9. D-1（正）居住区周边有幼儿园、托儿所、中小学、养老服务设施、卫生服务中心、商业服务设施等配置完善，便捷通达 [单选题] *

| 喜欢 | ○ | ○ | ○ | ○ | ○ | 不喜欢 |
| --- | --- | --- | --- | --- | --- | --- |

10. D-1（反）居住区周边公共服务设施（如幼儿园、托儿所、中小学、养老服务设施、卫生服务中心、商业服务设施等）配置不够完善，到达不太方便 [单选题] *

| 喜欢 | ○ | ○ | ○ | ○ | ○ | 不喜欢 |
| --- | --- | --- | --- | --- | --- | --- |

11. D-2（正）城区内设置公共开放空间供居民活动 [单选题] *

| 喜欢 | ○ | ○ | ○ | ○ | ○ | 不喜欢 |
| --- | --- | --- | --- | --- | --- | --- |

12. D-2（反）城区内无公共开放空间供居民活动 [单选题] *

| 喜欢 | ○ | ○ | ○ | ○ | ○ | 不喜欢 |
| --- | --- | --- | --- | --- | --- | --- |

13. D-3（正）城区内保有一定规模、布局合理的生态用地和城市绿地 [单选题] *

| 喜欢 | ○ | ○ | ○ | ○ | ○ | 不喜欢 |
| --- | --- | --- | --- | --- | --- | --- |

14. D-3（反）城区内生态用地和城市绿地较少，布局不够合理 [单选题] *

| 喜欢 | ○ | ○ | ○ | ○ | ○ | 不喜欢 |
| --- | --- | --- | --- | --- | --- | --- |

15. D-4（正）城区实施立体绿化，各类园林绿地养护管理良好，城区绿化覆盖率较高 [单选题] *

| 喜欢 | ○ | ○ | ○ | ○ | ○ | 不喜欢 |
| --- | --- | --- | --- | --- | --- | --- |

16. D-4（反）城区未实施立体绿化，各类园林绿地养护管理较差，城区绿化覆盖率较低 [单选题] *

| 喜欢 | ○ | ○ | ○ | ○ | ○ | 不喜欢 |
| --- | --- | --- | --- | --- | --- | --- |

17. D-5（正）实施海绵城市建设，暴雨不发生城市内涝 [单选题] *

| 喜欢 | ○ | ○ | ○ | ○ | ○ | 不喜欢 |
| --- | --- | --- | --- | --- | --- | --- |

18. D-5（反）海绵城市建设不到位，暴雨易发生城市内涝 [单选题] *

| 喜欢 | ○ | ○ | ○ | ○ | ○ | 不喜欢 |
| --- | --- | --- | --- | --- | --- | --- |

19. D-6（正）不出现土壤污染情况或完成土壤污染治理并达标 [单选题] *

| 喜欢 | ○ | ○ | ○ | ○ | ○ | 不喜欢 |
| --- | --- | --- | --- | --- | --- | --- |

20. D-6（反）出现土壤污染情况，土壤污染治理未达标 [单选题] *

| 喜欢 | ○ | ○ | ○ | ○ | ○ | 不喜欢 |
| --- | --- | --- | --- | --- | --- | --- |

21. D-7（正）市政生活用水质量达到国家标准 [单选题] *

| 喜欢 | ○ | ○ | ○ | ○ | ○ | 不喜欢 |
| --- | --- | --- | --- | --- | --- | --- |

22. D-7（反）市政生活用水质量未达到国家标准 [单选题] *

| 喜欢 | ○ | ○ | ○ | ○ | ○ | 不喜欢 |
| --- | --- | --- | --- | --- | --- | --- |

23. D-8（正）全年空气质量良好。优良日达到240天以上，PM2.5平均浓度达标天数达到200天以上 [单选题] *

| 喜欢 | ○ | ○ | ○ | ○ | ○ | 不喜欢 |
| --- | --- | --- | --- | --- | --- | --- |

24. D-8（反）全年空气质量较差。优良日240天以下，PM2.5平均浓度达标天数在200天以下 [单选题] *

| 喜欢 | ○ | ○ | ○ | ○ | ○ | 不喜欢 |
| --- | --- | --- | --- | --- | --- | --- |

25. D-9（正）区域环境噪声质量满足国家标准，较少出现室外噪声超标的情况 [单选题] *

| 喜欢 | ○ | ○ | ○ | ○ | ○ | 不喜欢 |
| --- | --- | --- | --- | --- | --- | --- |

26. D-9（反）区域环境噪声质量不满足国家标准，经常出现室外噪声超标的情况 [单选题] *

| 喜欢 | ○ | ○ | ○ | ○ | ○ | 不喜欢 |
| --- | --- | --- | --- | --- | --- | --- |

27. D-10（正）垃圾分类收集、密闭化运输、有效处理 [单选题] *

| 喜欢 | ○ | ○ | ○ | ○ | ○ | 不喜欢 |
| --- | --- | --- | --- | --- | --- | --- |

28. D-10（反）垃圾未分类收集，未采取密闭化运输的措施，垃圾处理措施不到位，垃圾房有异味传出 [单选题] *

| 喜欢 | ○ | ○ | ○ | ○ | ○ | 不喜欢 |
| --- | --- | --- | --- | --- | --- | --- |

29. D-11（正）对于新建建筑，购买获得绿色建筑认证的绿色住宅建筑或者入驻绿色办公楼工作 [单选题] *

| 喜欢 | ○ | ○ | ○ | ○ | ○ | 不喜欢 |
| --- | --- | --- | --- | --- | --- | --- |

30. D-11（反）拟选择购买的新建住宅或者工作要入驻的新建办公楼不是绿色建筑 [单选题] *

| 喜欢 | ○ | ○ | ○ | ○ | ○ | 不喜欢 |
| --- | --- | --- | --- | --- | --- | --- |

31. D-12（正）已经居住的住宅或者工作的办公楼进行绿色改造，达到国家绿色建筑的要求 [单选题] *

| 喜欢 | ○ | ○ | ○ | ○ | ○ | 不喜欢 |
| --- | --- | --- | --- | --- | --- | --- |

32. D-12（反）已经居住的住宅或者工作的办公楼未进行绿色改造，且未达到国家绿色建筑的要求 [单选题] *

| 喜欢 | ○ | ○ | ○ | ○ | ○ | 不喜欢 |
| --- | --- | --- | --- | --- | --- | --- |

33. D-13（正）正在建设的项目达到绿色施工的要求，降低对周边环境的污染和影响 [单选题] *

| 喜欢 | ○ | ○ | ○ | ○ | ○ | 不喜欢 |
| --- | --- | --- | --- | --- | --- | --- |

34. D-13（反）正在建设的项目未达到绿色施工的要求，对周边环境造成了污染和影响 [单选题] *

| 喜欢 | ○ | ○ | ○ | ○ | ○ | 不喜欢 |
| --- | --- | --- | --- | --- | --- | --- |

35. D-14（正）已经居住的住宅或者工作的办公楼采用绿色化运营，在降低运营费用的同时提供良好的建筑环境、优质的服务 [单选题] *

| 喜欢 | ○ | ○ | ○ | ○ | ○ | 不喜欢 |
| --- | --- | --- | --- | --- | --- | --- |

36. D-14（反）已经居住的住宅或者工作的办公楼未采用绿色化运营，运营费用较高，建筑环境与服务质量较差 [单选题] *

| 喜欢 | ○ | ○ | ○ | ○ | ○ | 不喜欢 |
| --- | --- | --- | --- | --- | --- | --- |

37. D-15（正）对于用热（冷）量进行计量收费 [单选题] *

| 喜欢 | ○ | ○ | ○ | ○ | ○ | 不喜欢 |
| --- | --- | --- | --- | --- | --- | --- |

38. D-15（反）对于用热（冷）量不进行计量收费，采用面积收费 [单选题] *

| 喜欢 | ○ | ○ | ○ | ○ | ○ | 不喜欢 |
| --- | --- | --- | --- | --- | --- | --- |

39. D-16（正）道路照明、景观照明、交通信号灯等采用高效灯具和光源，市政给水排水的水泵及相关设备等采用高效设备 [单选题] *

| 喜欢 | ○ | ○ | ○ | ○ | ○ | 不喜欢 |
| --- | --- | --- | --- | --- | --- | --- |

40. D-16（反）道路照明、景观照明、交通信号灯等未采用高效灯具和光源，市政给水排水的水泵及相关设备等未采用高效设备 [单选题] *

| 喜欢 | ○ | ○ | ○ | ○ | ○ | 不喜欢 |
| --- | --- | --- | --- | --- | --- | --- |

41. D-17（正）居住或工作的建筑具有利用再生水（中水）的条件 [单选题] *

| 喜欢 | ○ | ○ | ○ | ○ | ○ | 不喜欢 |
| --- | --- | --- | --- | --- | --- | --- |

42. D-17（反）居住或工作的建筑未采用再生水（中水），全部采用自来水 [单选题] *

| 喜欢 | ○ | ○ | ○ | ○ | ○ | 不喜欢 |
| --- | --- | --- | --- | --- | --- | --- |

43. D-18（正）公交站点覆盖率高，设置公交专用道，公交系统具有人性化服务设施（如导向、无障碍通道、遮阳、座椅等设施） [单选题] *

| 喜欢 | ○ | ○ | ○ | ○ | ○ | 不喜欢 |
| --- | --- | --- | --- | --- | --- | --- |

44. D-18（反）公交站点覆盖率低，未设置公交专用道，公交系统服务设施（如导向、无障碍通道、遮阳、座椅等）配置不够完善 [单选题] *

| 喜欢 | ○ | ○ | ○ | ○ | ○ | 不喜欢 |
| --- | --- | --- | --- | --- | --- | --- |

45. D-19（正）城区自行车道连续，具有合理宽度形成林荫路，具备完善的道路配套设施（如导向标识、安全、休息、环卫等设施） [单选题] *

| 喜欢 | ○ | ○ | ○ | ○ | ○ | 不喜欢 |
| --- | --- | --- | --- | --- | --- | --- |

46. D-19（反）城区自行车道不连续，自行车道未形成林荫路，道路配套设施（如导向标识、安全、休息、环卫等）配置不够完善 [单选题] *

| 喜欢 | ○ | ○ | ○ | ○ | ○ | 不喜欢 |
| --- | --- | --- | --- | --- | --- | --- |

47. D-20（正）步行系统连续，与周边功能、环境、景观、公共空间相结合，具有完善的配套设施（如照明、导向标识、安全、休息、环卫等设施） [单选题] *

| 喜欢 | ○ | ○ | ○ | ○ | ○ | 不喜欢 |
| --- | --- | --- | --- | --- | --- | --- |

48. D-20（反）步行系统不连续，与周边功能、环境、景观、公共空间结合的不好，步行系统配套设施（如照明、导向标识、安全、休息、环卫等）配置不够完善 [单选题] *

| 喜欢 | ○ | ○ | ○ | ○ | ○ | 不喜欢 |
| --- | --- | --- | --- | --- | --- | --- |

49. D-21（正）住宅、公共建筑、公共停车场配建电动车充电设施 [单选题] *

| 喜欢 | ○ | ○ | ○ | ○ | ○ | 不喜欢 |
| --- | --- | --- | --- | --- | --- | --- |

50. D-21（反）住宅、公共建筑、公共停车场未配建电动车充电设施，或充电设施很少 [单选题] *

| 喜欢 | ○ | ○ | ○ | ○ | ○ | 不喜欢 |
| --- | --- | --- | --- | --- | --- | --- |

51. D-22（正）地下停车场、公共停车场设置充足的停车位，且停车资源高效利用，停车信息及时通畅 [单选题] *

| 喜欢 | ○ | ○ | ○ | ○ | ○ | 不喜欢 |
| --- | --- | --- | --- | --- | --- | --- |

52. D-22（反）地下停车场、公共停车场停车位不足，且停车资源存在浪费闲置的情况，停车信息获取不及时不充分 [单选题] *

| 喜欢 | ○ | ○ | ○ | ○ | ○ | 不喜欢 |
| --- | --- | --- | --- | --- | --- | --- |

53. D-23（正）社区养老服务网络健全，养老床位充足 [单选题] *

| 喜欢 | ○ | ○ | ○ | ○ | ○ | 不喜欢 |
| --- | --- | --- | --- | --- | --- | --- |

54. D-23（反）社区养老服务网络不健全，养老床位不足 [单选题] *

| 喜欢 | ○ | ○ | ○ | ○ | ○ | 不喜欢 |
| --- | --- | --- | --- | --- | --- | --- |

55. D-24（正）过街天桥和隧道设置无障碍电梯或扶梯，人行横道设置盲人过街语音信号灯，设置夜间行人按钮式信号灯 [单选题] *

| 喜欢 | ○ | ○ | ○ | ○ | ○ | 不喜欢 |
| --- | --- | --- | --- | --- | --- | --- |

56. D-24（反）过街天桥和隧道未设置无障碍电梯或扶梯，人行横道未设置盲人过街语音信号灯，未设置夜间行人按钮式信号灯 [单选题] *

| 喜欢 | ○ | ○ | ○ | ○ | ○ | 不喜欢 |
| --- | --- | --- | --- | --- | --- | --- |

57. D-25（正）制定优惠措施鼓励居民购置节能家电、节水器具 [单选题] *

| 喜欢 | ○ | ○ | ○ | ○ | ○ | 不喜欢 |
| --- | --- | --- | --- | --- | --- | --- |

58. D-25（反）未制定优惠措施鼓励居民购置节能家电、节水器具 [单选题] *

| 喜欢 | ○ | ○ | ○ | ○ | ○ | 不喜欢 |
| --- | --- | --- | --- | --- | --- | --- |

三、对需求重要度的测评
以下问题针对您对于绿色生态城区某项需求的重要程度进行测评。
按照您认为某项需求的重要程度，包含5个选择：1分-非常不重要、2分-不太重要、3分-一般重要、4分-比较重要、5分-非常重要。分数越高代表重要程度越高。

59. D1-居住区周边有幼儿园、托儿所、中小学、养老服务设施、卫生服务中心、商业服务设施等配置完善，便捷通达[矩阵量表题] *

|  | 非常不重要 | 不太重要 | 一般重要 | 比较重要 | 非常重要 |
| --- | --- | --- | --- | --- | --- |
| 重要程度 | ○ | ○ | ○ | ○ | ○ |

60. D2-城区内设置公共开放空间供居民活动[矩阵量表题] *

|  | 非常不重要 | 不太重要 | 一般重要 | 比较重要 | 非常重要 |
| --- | --- | --- | --- | --- | --- |
| 重要程度 | ○ | ○ | ○ | ○ | ○ |

61. D3-城区内保有一定规模、布局合理的生态用地和城市绿地[矩阵量表题] *

|  | 非常不重要 | 不太重要 | 一般重要 | 比较重要 | 非常重要 |
| --- | --- | --- | --- | --- | --- |
| 重要程度 | ○ | ○ | ○ | ○ | ○ |

62. D4-城区实施立体绿化，各类园林绿地养护管理良好，城区绿化覆盖率较高[矩阵量表题] *

|  | 非常不重要 | 不太重要 | 一般重要 | 比较重要 | 非常重要 |
| --- | --- | --- | --- | --- | --- |
| 重要程度 | ○ | ○ | ○ | ○ | ○ |

63. D5-实施海绵城市建设，暴雨不发生城市内涝[矩阵量表题] *

|  | 非常不重要 | 不太重要 | 一般重要 | 比较重要 | 非常重要 |
| --- | --- | --- | --- | --- | --- |
| 重要程度 | ○ | ○ | ○ | ○ | ○ |

64. D6-不出现土壤污染情况或完成土壤污染治理并达标[矩阵量表题] *

|  | 非常不重要 | 不太重要 | 一般重要 | 比较重要 | 非常重要 |
| --- | --- | --- | --- | --- | --- |
| 重要程度 | ○ | ○ | ○ | ○ | ○ |

65. D7-市政生活用水质量达到国家标准[矩阵量表题] *

|  | 非常不重要 | 不太重要 | 一般重要 | 比较重要 | 非常重要 |
| --- | --- | --- | --- | --- | --- |
| 重要程度 | ○ | ○ | ○ | ○ | ○ |

66. D8-全年空气质量良好。优良日达到240天以上，PM2.5平均浓度达标天数达到200天以上[矩阵量表题] *

|  | 非常不重要 | 不太重要 | 一般重要 | 比较重要 | 非常重要 |
| --- | --- | --- | --- | --- | --- |
| 重要程度 | ○ | ○ | ○ | ○ | ○ |

67. D9-区域环境噪声质量满足国家标准，较少出现室外噪声超标的情况[矩阵量表题] *

|  | 非常不重要 | 不太重要 | 一般重要 | 比较重要 | 非常重要 |
| --- | --- | --- | --- | --- | --- |
| 重要程度 | ○ | ○ | ○ | ○ | ○ |

68. D10-垃圾分类收集、密闭化运输、有效处理[矩阵量表题] *

|  | 非常不重要 | 不太重要 | 一般重要 | 比较重要 | 非常重要 |
| --- | --- | --- | --- | --- | --- |
| 重要程度 | ○ | ○ | ○ | ○ | ○ |

69. D11-对于新建建筑，购买获得绿色建筑认证的绿色住宅建筑或者入驻绿色办公楼工作[矩阵量表题] *

|  | 非常不重要 | 不太重要 | 一般重要 | 比较重要 | 非常重要 |
| --- | --- | --- | --- | --- | --- |
| 重要程度 | ○ | ○ | ○ | ○ | ○ |

70. D12-已经居住的住宅或者工作的办公楼进行绿色改造，达到国家绿色建筑的要求[矩阵量表题] *

|  | 非常不重要 | 不太重要 | 一般重要 | 比较重要 | 非常重要 |
| --- | --- | --- | --- | --- | --- |
| 重要程度 | ○ | ○ | ○ | ○ | ○ |

71. D13-正在建设的项目达到绿色施工的要求，降低对周边环境的污染和影响[矩阵量表题] *

|  | 非常不重要 | 不太重要 | 一般重要 | 比较重要 | 非常重要 |
| --- | --- | --- | --- | --- | --- |
| 重要程度 | ○ | ○ | ○ | ○ | ○ |

72. D14-已经居住的住宅或者工作的办公楼采用绿色化运营，在降低运营费用的同时提供良好的建筑环境、优质的服务[矩阵量表题] *

|  | 非常不重要 | 不太重要 | 一般重要 | 比较重要 | 非常重要 |
| --- | --- | --- | --- | --- | --- |
| 重要程度 | ○ | ○ | ○ | ○ | ○ |

73. D15-对于用热（冷）量进行计量收费[矩阵量表题] *

|  | 非常不重要 | 不太重要 | 一般重要 | 比较重要 | 非常重要 |
| --- | --- | --- | --- | --- | --- |
| 重要程度 | ○ | ○ | ○ | ○ | ○ |

74. D16-道路照明、景观照明、交通信号灯等采用高效灯具和光源，市政给水排水的水泵及相关设备等采用高效设备[矩阵量表题] *

|  | 非常不重要 | 不太重要 | 一般重要 | 比较重要 | 非常重要 |
| --- | --- | --- | --- | --- | --- |
| 重要程度 | ○ | ○ | ○ | ○ | ○ |

75. D17-居住或工作的建筑具有利用再生水（中水）的条件[矩阵量表题] *

|  | 非常不重要 | 不太重要 | 一般重要 | 比较重要 | 非常重要 |
| --- | --- | --- | --- | --- | --- |
| 重要程度 | ○ | ○ | ○ | ○ | ○ |

76. D18-公交站点覆盖率高，设置公交专用道，公交系统具有人性化服务设施（如导向、无障碍通道、遮阳、座椅等设施）[矩阵量表题] *

|  | 非常不重要 | 不太重要 | 一般重要 | 比较重要 | 非常重要 |
| --- | --- | --- | --- | --- | --- |
| 重要程度 | ○ | ○ | ○ | ○ | ○ |

77. D19-城区自行车道连续，具有合理宽度形成林荫路，具备完善的道路配套设施（如导向标识、安全、休息、环卫等设施）[矩阵量表题] *

|  | 非常不重要 | 不太重要 | 一般重要 | 比较重要 | 非常重要 |
| --- | --- | --- | --- | --- | --- |
| 重要程度 | ○ | ○ | ○ | ○ | ○ |

78. D20-步行系统连续，与周边功能、环境、景观、公共空间相结合，具有完善的配套设施（如照明、导向标识、安全、休息、环卫等设施）[矩阵量表题] *

|  | 非常不重要 | 不太重要 | 一般重要 | 比较重要 | 非常重要 |
| --- | --- | --- | --- | --- | --- |
| 重要程度 | ○ | ○ | ○ | ○ | ○ |

79. D21-住宅、公共建筑、公共停车场配建电动车充电设施[矩阵量表题] *

|  | 非常不重要 | 不太重要 | 一般重要 | 比较重要 | 非常重要 |
| --- | --- | --- | --- | --- | --- |
| 重要程度 | ○ | ○ | ○ | ○ | ○ |

80. D22-地下停车场、公共停车场设置充足的停车位，且停车资源高效利用，停车信息及时通畅[矩阵量表题] *

|  | 非常不重要 | 不太重要 | 一般重要 | 比较重要 | 非常重要 |
| --- | --- | --- | --- | --- | --- |
| 重要程度 | ○ | ○ | ○ | ○ | ○ |

81. D23-社区养老服务网络健全，养老床位充足[矩阵量表题] *

|  | 非常不重要 | 不太重要 | 一般重要 | 比较重要 | 非常重要 |
| --- | --- | --- | --- | --- | --- |
| 重要程度 | ○ | ○ | ○ | ○ | ○ |

82. D24-过街天桥和隧道设置无障碍电梯或扶梯，人行横道设置盲人过街语音信号灯，设置夜间行人按钮式信号灯[矩阵量表题] *

|  | 非常不重要 | 不太重要 | 一般重要 | 比较重要 | 非常重要 |
| --- | --- | --- | --- | --- | --- |
| 重要程度 | ○ | ○ | ○ | ○ | ○ |

83. D25-制定优惠措施鼓励居民购置节能家电、节水器具[矩阵量表题] *

|  | 非常不重要 | 不太重要 | 一般重要 | 比较重要 | 非常重要 |
| --- | --- | --- | --- | --- | --- |
| 重要程度 | ○ | ○ | ○ | ○ | ○ |

**问卷到此结束，衷心感谢您的配合，祝您工作、生活愉快！**
